# Angiofibrome orbitaire à cellules géantes : à propos d'un cas

**DOI:** 10.11604/pamj.2014.17.51.3582

**Published:** 2014-01-23

**Authors:** Kamal Loutfi Nuiakh, Hicham Tahri

**Affiliations:** 1 Service d'Ophtalmologie, CHU Hassan II Fès, Maroc

**Keywords:** Angiofibrome, orbite, cellules géantes, angiofibroma, eye-socket, giant cells

## Image en medicine

Une jeune âgée de 16 ans, sans antécédent tumoral ou inflammatoire, présentait depuis 6 mois une exophtalmie unilatérale droite non axile irréductible sans signes inflammatoires (A), d'installation rapidement progressive, associée à une limitation du muscle droit externe homolatérale. La tomodensitométrie objectivait une tumeur hétérogène au niveau de l'angle supéro-externe droit, avec des calcifications intra-tissulaires et des érosions osseuses (B). L’étude histopathologique montrait la présence de multiples cellules géantes multi-nucléées disposées en couronne, avec un immunomarquage positif par CD34 associées à un tissu fibreux parsemé de nombreux capillaires, compatible avec un angiofibrome à cellules géantes (C, D). Après un recul post-opératoire de 10 mois, on n'a pas noté de récidive. L'angiofibrome à cellules géantes est une tumeur bénigne fibroblastique très rare, qui prédomine au niveau orbitaire et des tissus mous de la face. L'aspect radio-clinique est souvent trompeur, pouvant simuler une tumeur agressive. Les caractéristiques histopathologiques sont très évocatrices du diagnostic. L’évolution n'est pas dénuée de récidives. Cette tumeur représente un défi diagnostique devant les aspects trompeurs, et un défi thérapeutique devant la difficulté de l'exérèse chirurgicale complète.

**Figure 1:**
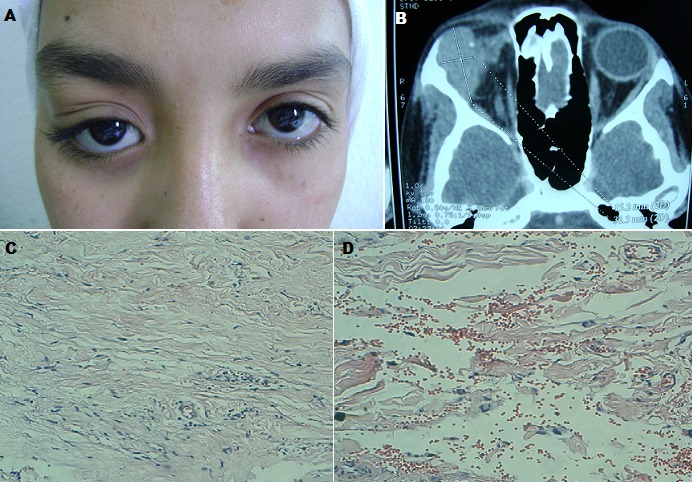
A) une exophtalmie unilatérale droite; B) Coupe axile de la TDM orbitaire montrant une masse tissulaire occupant l'angle supéro-externe de l'orbite droit, associée à des calcifications intra-tumorales; C) Prolifération tumorale de cellularité modérée faite de cellules allongées baignant dans un stroma riche en collagène(HEx20); D) La vascularisation est proéminente : vaisseaux congestifs de taille variable avec présence d'espaces pseudo-vasculaires entourés par des cellules géantes multi-nucléées (HEx20)

